# Floristic Diversity and Distribution Pattern of Plant Communities along Altitudinal Gradient in Sangla Valley, Northwest Himalaya

**DOI:** 10.1155/2014/264878

**Published:** 2014-10-14

**Authors:** Pankaj Sharma, J. C. Rana, Usha Devi, S. S. Randhawa, Rajesh Kumar

**Affiliations:** ^1^National Bureau of Plant Genetic Resources, Regional Station, Phagli, Shimla 171 004, India; ^2^State Council for Science, Technology and Environment, Shimla 171 009, India; ^3^School of Basic Sciences and Research, Sharda University, Knowledge Park III, Greater Noida, Gautam Budh Nagar 201306, India

## Abstract

Himalayas are globally important biodiversity hotspots and are facing rapid loss in floristic diversity and changing pattern of vegetation due to various biotic and abiotic factors. This has necessitated the qualitative and quantitative assessment of vegetation here. The present study was conducted in Sangla Valley of northwest Himalaya aiming to assess the structure of vegetation and its trend in the valley along the altitudinal gradient. In the forest and alpine zones of the valley, 15 communities were recorded. Study revealed 320 species belonging to 199 genera and 75 families. Asteraceae, Rosaceae, Apiaceae, and Ranunculaceae were dominant. Among genera, *Artemisia* followed by *Polygonum*, *Saussurea*, *Berberis*, and *Thalictrum* were dominant. Tree and shrub's density ranged from 205 to 600 and from 105 to 1030 individual per hectare, respectively, whereas herbs ranged from 22.08 to 78.95 individual/m^2^. Nearly 182 species were native to the Himalaya. Maximum altitudinal distribution of few selected climate sensitive species was found to be highest in northeast and north aspects. This study gives an insight into the floristic diversity and community structure of the fragile Sangla Valley which was hitherto not available.

## 1. Introduction

Himalayas comprised of earth's most multifaceted and diverse montane ecosystems, characterized by a harsh climate, a strong degree of seasonality, and a high diversity of both plant communities and species [1, 2]. These are geodynamic young mountains and have been recognized as one of the globally important biodiversity hotspots [3, 4]. In these mountains presence of often sharp environmental gradients due to rapid geoclimatic variations generate diverse vegetation and community types having high plant species diversity [5]. Here a wide range of altitude, rainfall, climate, geological conditions, river systems, and topography have given rise to an immense diversity of ecosystems and ultimately to immense biological diversity.

The structure, composition, and vegetative functions are most significant ecological attributes of a particular ecosystem, which show variations in response to environmental as well as anthropogenic variables [6–8]. Major threats to ecosystems and biodiversity are habitat loss and fragmentation, overexploitation, pollution, invasions of alien species, and global climate change [9] with disruption of community structure.

The vegetation distribution pattern, communities, and population dynamics in high altitude arid areas of the fragile Himalaya have seldom given the due attention by researchers and are hence poorly understood. Furthermore, altitude and aspect are the major topographic factors that control the distribution patterns of vegetation in mountain areas. These factors determine the microclimate and thus the distribution of vegetation in the mountain areas [10]. The anthropogenic pressures, heavy grazing, and the natural calamities have led to degradation of natural habitats of many species. Such practices are discouraging the moisture loving native species and promoting the hardy nonnative exotic species having little value for the local ecosystem [11].

The rapid loss in floristic diversity and changing pattern of vegetation due to various biotic and abiotic factors have necessitated the qualitative and quantitative assessment of vegetation. However numbers of studies on community dynamics and phytogeographic affinities have been conducted qualitatively [12–17] as well as quantitatively [5, 18–24] and in northwest Himalaya [4, 6, 25–27] in particular as well. Nevertheless, a very few studies incorporating composition, structural and functional diversity, and nativity of the biodiversity have been carried out in northwest Himalaya [28–31]. But not many studies that give detailed account of floristic diversity of Kinnaur [5], which forms a typical geographical entity of Himachal Pradesh and Sangla Valley in particular, are available till now. Therefore, this work is emphasized to study floristic composition; to assess the community structure of the vegetation by different phytosociological methods and phytogeographic affinities of the species; and to study the vegetation pattern in the different aspects of the SV along an altitudinal gradient.

## 2. Materials and Methods

### 2.1. Physiographic Features of the Study Area

**Figure 1:**
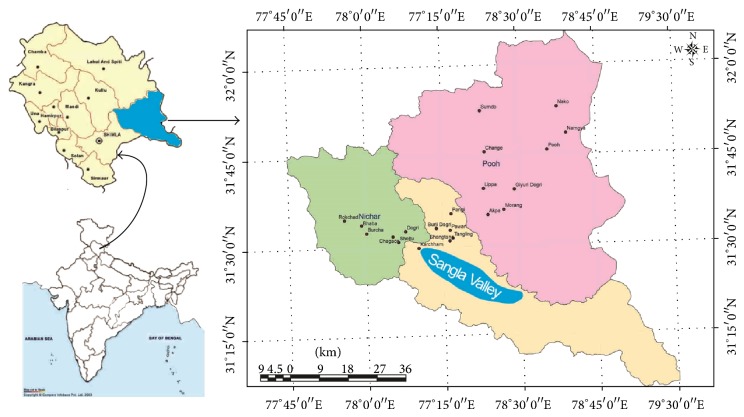
Map of the study area.

The study area is commonly known as Sangla Valley (hereafter, SV) and situated at 31°31′– 36′N and 77°20′−27′E along the Baspa River that flows through the middle of valley (Figure 1). The valley is oriented from southeast to northwest directions. This is one of the most ecologically fragile biogeographical zones [32] and inhabited by indigenous tribal communities having Mongolian features and Buddhist religion and culture. It is surrounded by high mountains with elevation ranging from 1800 to 5480 m above mean sea level. The upper ranges of the valley are highly glaciered and receive most of its water through dry precipitation (snow) in winters from November to April. The vegetation of the valley is temperate; subalpine and alpine types and forests are dominated by* Pinus wallichiana*,* Betula utilis*,* Abies pindrow*, and* Cedrus deodara* communities. The livelihood of communities is based on agrihorticultural activities, which are generally performed after snow melt in April to October. The communities have close affinity with plant resources not only to meet their basic requirements like food, fodder, fuel, health, and shelter but also to perform several religious and cultural rituals. Though the environment of the valley is very close to nature, several anthropogenic activities have successfully altered the natural and traditional agroecosystem of the valley. Several new climatic events such as increasing frequency of rains in July-August (150–200 mm in 1980s to 465 mm in 2012), rising temperature, frost, and fog are being witnessed more frequently than two decades ago.

### 2.2. Sampling Plot and Estimation Design

Representative plots of 50 × 50 m were selected in different aspects and habitats. 10 quadrats of 10 × 10 m for trees, 20 quadrats of 5 × 5 m for shrubs, and 20 quadrats of 1 × 1 m for herbs were randomly laid within the plot. Plots were selected based on different topographical features such as habitat types, altitude, aspects, slope, and different vegetation types (Table 1). The habitats were identified based on the physical characters and dominance of the vegetation. The plots facing high anthropogenic pressure were considered as degraded habitats and sites having closed canopy with high percent of humus and moisture were considered as moist habitat whereas those of low percent of the same were considered as dry habitat. The site having >50% boulders of the ground cover were considered as bouldery habitat. Geographical coordinates of the sites were taken with the help of Global Positioning System (GPS). Slope was measured with the help of Abney's level.

Sites were selected in each and every aspect between 1950 and 4500 m for the field study and analysis of floristic diversity. In various representative ecoclimatic zones of SV, 34 plots were sampled.

### 2.3. Data Analysis

The SV, which falls under cold arid zone of Himachal Pradesh, is diverse and rich in species. Communities were identified based on the importance value index and calculated as the sum of relative frequency, relative density, and relative basal area/relative abundance. For diversity index, Shannon-Wiener information index [33] was used. Species richness was considered as the total number of species in a growth form. For the collection and analysis of data standard ecological methods [20, 24, 34–39] were followed. Vegetation was analytically computed following [39, 40]. The taxa of Himalayan origin were considered as native and all others as nonnative. During qualitative assessment, rapid surveys and samplings were done in each season and taxa identified on spot and with the help of floras and research papers [12–14, 40–43].

## 3. Results and Discussion

Plots accessed were 34 in number and a total 15 communities (9 tree, 5 shrub, and 1 herb communities) were delineated based on importance value index for the tree communities and relative density for the shrub and herb communities.

### 3.1. Vegetational Analysis

The different habitats covered during the study were shady moist, rocky, bouldery, dry, alpine meadow, riverine, moraines, and so forth. Among these, shady moist (12) followed by dry habitats (9) represented maximum sites. Because of unique topography and different altitudinal zones of western Himalaya, vegetation varies from aspect to aspect. The sites were selected in every accessible habitat and aspect along an altitudinal gradient. North and northeastern aspects represented maximum sites (8 each) followed by southwest and northwest (5 each) (Table 1).

Within the communities so delineated, we recorded 320 species of plants belonging to 199 genera and 75 families. Dominant group reported was angiosperms, (68 families, 190 genera, and 302 species); gymnosperms represented by 4 families, 7 genera, and 13 species and pteridophytes by 4 families, 3 genera, and 5 species. They were distributed in different life forms, that is, trees (29 spp.), shrubs (43 spp.), and herbs (248 spp.) (Table 2).

Among the angiosperm families, Asteraceae (49 spp.); Rosaceae (21 spp.); Apiaceae (20 spp.); and Ranunculaceae (18 spp.) were dominant. Among genera,* Artemisia *and* Polygonum *(7 spp.);* Saussurea *(6 spp.);* Berberis *and* Thalictrum *(5 spp.), and* Geranium*,* Juniperus*,* Nepeta*,* Potentilla*,* Poa*,* Rosa*, and* Salix *(4 spp. each) were dominant genera. Twenty-eight (28) families were monotypic and represented only by one species. The occurrence of 320 species in the quantified area indicates that its environmental conditions, particularly shady moist and forest habitats, are suitable for the growth and development of species. Amongst the communities,* P. wallichiana* community represented maximum sites (6 sites), followed by* B. utilis* (5 sites);* B. utilis*-*P. wallichiana* mixed (4 sites);* C. deodara *and* P. gerardiana *(3 sites, each);* R. anthopogon*,* H. salicifolia*, and* R. anthopogon-S. caliculata* mixed (2 sites, each), and the rest of the communities were represented by one site only. Dominance of the Asteraceae in SV is also validated by floras of Lahaul-Spiti, Himachal Pradesh, in high altitude regions of western Himalaya [13, 42]. The affinity of vegetation towards the flora of the Lahaul-Spiti Valley and Bhaba Valley [5, 42] is apparent by the presence of similar dominant families. Moreover, the major part of the valley is covered with snow throughout the year. Presence of number of herbaceous families (namely, Apiaceae, Brassicaceae, Ranunculaceae, Rosaceae, Polygonaceae, and Scrophulariaceae) is attributed to the temperate and alpine nature of the area. Lesser Pteridophytes in the area may be attributed to the more exposed arid nature of the valley with low broad leaf forest cover and moisture. Nonetheless, as a whole, the high diversity and richness of the species in the SV indicate the high conservation value of the area. Occurrences of 320 species in the 15 identified communities of 34 quantified plots validate this.

### 3.2. Communities: Composition and Structure

Total tree density ranged from 205 to 600 no./ha (number per hectare) and total basal area from 8.70 to 42.41 m^2^/ha. Shrubs and herbs densities ranged from 105.0 to 1030.00 no./ha and from 22.08 to 48.73 no./m^2^_,_ respectively. Shrub density is maximum in* C. deodara-P. smithiana* mixed community and herbs density is maximum in* Poa alpina-Agrostis stolonifera-Bistorta affinis-Aconitum violaceum *community. Among five major shrub communities,* Spiraea canescens-Lonicera hypoleuca* mixed community has highest shrub and herb density, that is, 540.00 no./ha and 48.73 no./m^2^, respectively (Table 3). Tree density range is comparable to the other Himalayan studies [40, 44] and European temperate forests [45]. Similarly, shrub and herb's density ranges (105–1030 no./ha and 22.08–78.95 no./m^2^, resp.) are in compliance with the earlier studies of the Himalayan regions [46]. However the lower range of shrub densities in* R. anthopogon* communities (105 no./ha and 230 no./ha) in the valley is due to the presence of its scanty patches near the subalpine areas and often the rugged and arid and moraine topography.

### 3.3. Species Richness and Diversity Index (H′)

Species richness in identified communities ranged from 19 to 96. Among the communities, it was highest in* P. wallichiana *(96 spp.), followed by* B. utilis-P. wallichiana* mixed (80 spp.),* R. anthopogon-S. caliculata *mixed (52 spp.), and* P. gerardiana *(47 spp.) communities. Species diversity index for trees is maximum (1.28) for* C. deodara-P. smithiana* mixed community and minimum (0.0) for* Q. floribunda *and* P. wallichiana *communities; among shrubs it is maximum (2.38) for* P. wallichiana *community and minimum (0.40) for* Q. floribunda* community and for herbs it is maximum (4.01) for* P. wallichiana* community and minimum (2.49) for* Q. floribunda* community (Table 3).

The species richness (19–96) in the communities was higher than the earlier reported values [47, 48] but comparable to the [24, 49] from high altitude areas of Himalaya. The high richness of trees and shrubs may be due to diverse habitats and suitable edaphic and climatic factors supporting growth and survival of the species. The diversity index for trees (0.0–1.28) is within the reported value from the other Himalayan areas [40, 47, 49, 50]. In* P. gerardiana *community, it is the only tree species which is present, so its diversity index value is zero. The diversity of shrubs (0.40–2.38) is comparable to the previous records from the higher Himalaya and also from the lower parts (0.51–1.33) [46, 48, 50] and (0.74–3.14) reported by [40, 51] for subtropical and temperate forests. However for herbs diversity (2.49–4.01) it was higher than earlier records. The value of the total basal area was found to be maximum in* C. deodara* and* C. deodara-P. wallichiana* mixed communities (42.41 and 21.99 m^2^/ha, resp.) and average basal area is 17.23 m^2^/ha which is very low as compared to the other parts of the Himalaya where it is above 70 m^2^/ha [52–54]. This may be due to unscrupulous tree felling and logging pressure on the forest resources in addition to the other natural causes like heavy erratic rainfalls which leads to the massive landslides in the region.

### 3.4. Species Dominance in the Identified Communities

Among the various communities identified in SV, we figured out the maximum values of dominant species as 74.5% (*B. utilis*) and 19.9% (*P. wallichiana*) in* B. utilis* community; 80.2% (*C. deodara*) and 17.4% (*P. wallichiana*) in* C. deodara *community; 100% (*Q. floribunda*) in* Q. floribunda *community as it was the only tree species present in the community; 57.14% (*Pinus gerardiana*) and 38.8%(*Quercus floribunda*) in* Pinus gerardiana *community; 90.9% (*P. wallichiana*) and 8.1% (*C. deodara*) in* P. wallichiana *community; 43.1% (*B. utilis*) and 40.9% (*A. pindrow*) in* B. utilis*-*A. pindrow *mixed community; 62.5% (*B. utilis*) and 29.8% (*P. wallichiana*)in* B. utilis-P. wallichiana *mixed community; 38.3% (*C. deodara*) and 31.6% (*Picea smithiana*) in* C. deodara-P. smithiana *mixed community; and 52.5% (*C. deodara*) and 42.5% (*P. wallichiana*) in* C. deodara-P. wallichiana* mixed community (Table 4).

Among shrubs maximum density percentages were of the species* Berberis aristata*,* Berberis jaeschkeana*,* Cassiope fastigiata*,* Cotoneaster bacillaris*, Cotoneaster* microphyllus*,* Desmodium elegans*,* Hippophae salicifolia*,* Lonicera hypoleuca*,* Rhododendron campanulatum*,* Rabdosia rugosa*,* Rhododendron anthopogon*,* Rubus ellipticus*,* Salix calyculata*,* Sorbaria tomentosa*,* Spiraea canescens*, and so forth.* Rabdosia rugosa* (80.1%) and* Cotoneaster bacillaris* (7.5%) were having maximum and minimum density percentages in* Quercus floribunda* and* Cedrus deodara* communities, respectively. Among herbs assessed* Aconogonum molle*,* Agrostis stolonifera*,* Bistorta affinis*,* Bromus japonicas*,* Cannabis sativa*,* Conyza sumatrensis*,* Cynoglossum wallichii*,* Delphinium cashmerianum*,* Euphrasia officinalis*,* Fragaria nubicola*,* Impatiens thomsonii*,* Morina longifolia*,* Nepeta erecta*,* Origanum vulgare*,* Persicaria vivipara*,* Poa alpina*,* Polygonatum verticillatum*,* Thalictrum cultratum*,* Trifolium pratense*, and so forth were in abundance, hence showing comparatively more densities. Herbs having maximum density recorded were* Poa alpina* (25.3%) followed by* Cynoglossum wallichii* (23.4%) and* Fragaria nubicola* (19.5%) in* Poa alpina-Agrostis stolonifera-Bistorta affinis-Aconitum violaceum* mixed,* B. utilis-A. pindrow* mixed, and* Rhododendron anthopogon-Salix calyculata* mixed communities, respectively (Table 4).

Tree, shrub, and herb percentage covers within the communities showed a typical composition of the Himalayan region wherein shrubs* Rabdosia rugosa *and* Cotoneaster bacillaris *were having maximum and minimum density percentages, respectively, and herbs* Poa alpina, Cynoglossum wallichii*,and* Fragaria nubicola* were abundant. Dominance of these species might be due to their high adaptability in addition to their good capability to proliferate in the extreme climatic conditions of this part of western Himalaya.

### 3.5. Phytogeographic Affinities

**Figure 2:**
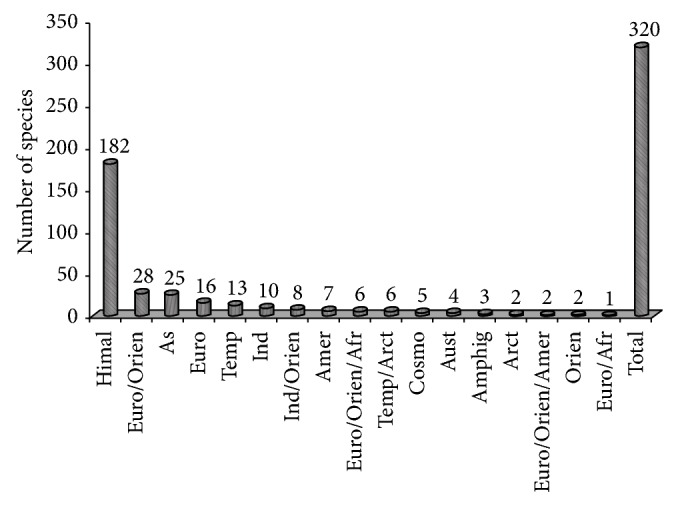
Species showing their biogeographic realms. Afr = Africa; Amer = America; Amphig = Amphigean; Arct = Arctic; As = Asia; Aust = Australia; Cosmo = Cosmopolitan; Euro = Europe; Himal = Himalaya; Ind = India; Orient = Oriental; and Temp = Temperate.

As a whole in all the communities, 182 species were native to the Himalayan region and the remaining were nonnatives as they are from different biogeographic domains of the world. The trend of nativity of plants occurring in SV was as follows: European/Oriental region (28) > Asia (25) > European region (16) > Temperate region (13) > Indian region (10) > India/Oriental region (8) > America (7) > European/Oriental/African and Temperate, Arctic (6 each) > Cosmopolitan (5) > Australian (4) > Amphigean (3) > Arctic, European/Oriental/American and Oriental (2 each) and European/African (1) (Figure 2).

**Figure 3:**
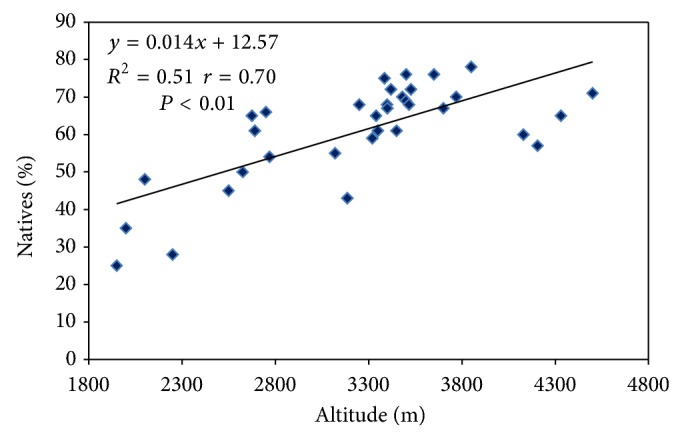
Native species along the altitude gradient.

Natives are the species which evolved naturally in a particular region before their human introduction. To prioritize a species or habitat of the region for conservation, status of a species as to whether it is native or introduced in a given area is required. Species invasions beyond their native range constitute a global driver of change as nonnative species threaten biodiversity and change ecosystem functioning [55]. Like in other parts of the Himachal Himalaya [29, 30] in SV also the percentage of native species increased with the altitude. There is a strong evidence from a scatter diagram that a positive linear relationship exists between the native species richness and altitude (*r* = 0.83, *P* < 0.01, *n* = 34) (Figure 3). The high percentage of the native species at higher elevations may be due to low anthropogenic pressure and severe climatic conditions compared to the lower elevations where high anthropogenic pressure and mild climatic conditions support the speciation of the nonnative species [30]. Regular monitoring of the habitats and populations of the native species facing high anthropogenic pressure even in higher altitude is essentially required, so that the adequate planning for their conservation and management could be done in time.

### 3.6. Altitudinal and Aspectwise Distribution of Species

Altitude and aspect are the most important determinants of vegetation distribution due to their direct impact on microclimate of the habitat [56, 57]. The Himalayan region has typical topography and environment where biodiversity varies from aspect to aspect and habitats of the communities [58].

**Figure 4:**
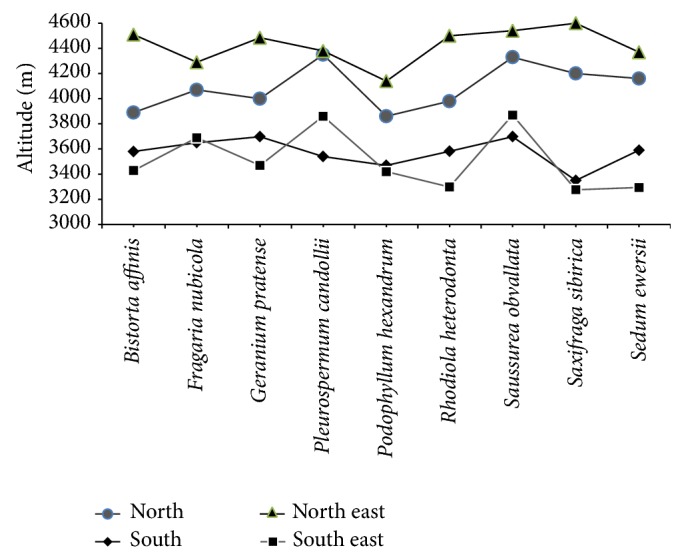
Distribution range of species along altitudinal gradient in different aspects.

Maximum altitudinal distribution of few selected climate sensitive species, namely,* Bistorta affinis*,* Fragaria nubicola*,* Geranium pratense*,* Pleurospermum candollei*,* Podophyllum hexandrum*,* Rhodiola heterodonta*,* Saussurea obvallata*,* Saxifraga sibrica*, and* Sedum ewersii*, was studied in the valley. It was found to be highest in northeast followed by north, south, and southeast aspects (Figure 4). Species like* Bistorta affinis* reaching up to 4510 m and 3890 m in northeast and north aspects, respectively, are restricted to 3580 m and 3429 m in south and southeast aspects, resepectively. Similar trend was seen with all other climate sesitive species in the region.

*P. wallichiana* showed the broadest range from 2100 to 3500 m and almost reaching the tree line along with* B. utilis*.

In this valley, northern and northeastern slopes have lower temperatures and higher soil and air moisture contents as compared to southern and other slopes at the same altitude due to less solar exposure and higher moisture content and evapotranspiration which is akin to the other Himalayan areas [59, 60]. In northern and northeastern slopes* B. utilis, A. pindrow*, and* P. wallichiana* were recorded at the altitude as low as 2200 m, whereas on the south and southeastern aspects their altitudinal range started from 2300 m.

### 3.7. Final Considerations

In northwestern Himalaya the high mountain plant communities support a rich biodiversity in terms of ecological indicator species and natives. They need a proper management against harsh climate and anthropogenic pressure for continued future sustainability. Regular monitoring using random sampling by quadrat method is suggested to understand the dynamics of the habitats and communities and accordingly plan for their management. The climate sensitive species are required to be regularly monitored for their phenological attributes so that the baseline data can be generated for future changes in the area. The information generated on these lines will provide a better insight about the present status of floristic diversity and help in developing adequate strategies and action plan for the management of such biodiversity-rich areas. The state and central government agencies are suggested to encourage the native species so that the ambient regional ecosystems are protected for the posterity. Further, for* in situ* conservation of the economically and ecologically important species, regular monitoring of the sites and complete protection of the habitats is suggested. In addition, seed germination protocols developed may be used for mass multiplication of the species and seedlings should be transplanted in comparable habitats so that viable population of the species can be maintained. Finally a pragmatic and ameliorative conservational approach which was hitherto absent in this part of the Himalaya needs to be implemented.

## Figures and Tables

**Table 1:** Physical Characteristics of the plots assessed in Sangla Valley.

S. No.	Altitude (m)	Habitat	Slope	Aspect	Latitude	Longitude
1	1950	Degraded	35°	S	31° 28.040 N	78° 11.209 E
2	2000	Dry	60°	NW	31° 28.853 N	78° 10.892 E
3	2100	Dry	60°	SW	31° 28.823 N	78° 10.962 E
4	2250	Dry	50°	S	31° 28.045 N	78° 11.111 E
5	2550	Shady Moist	20°	N	31° 25.025 N	78° 16.103 E
6	2625	Riverine	50°	NE	31° 24.854 N	78° 16.828 E
7	2675	Bouldery	40°	N	31° 25.061 N	78° 16.368 E
8	2690	Dry	15°	NE	31° 24.913 N	78° 16.085 E
9	2750	Shady Moist	10°	SW	31° 25.758 N	78° 16.746 E
10	2770	Shady Moist	45°	NE	31° 24.342 N	78° 18.038 E
11	3120	Bouldery	20°	NE	31° 23.039 N	78° 21.614 E
12	3185	Dry	30°	SW	31° 23.631 N	78° 21.370 E
13	3250	Dry	45°	S	31° 24.342 N	78° 18.038 E
14	3320	Dry	35°	SW	31° 21.286 N	78° 24.438 E
15	3340	Shady Moist	55°	SW	31° 23.840 N	78° 21.340 E
16	3350	Rocky	60°	S	31° 21.756 N	78° 24.029 E
17	3385	Dry	45°	N	31° 20.992 N	78° 26.287 E
18	3399	Dry	40°	N	31° 23.640 N	78° 21.558 E
19	3400	Shady Moist	40°	N	31° 20.955 N	78° 26.135 E
20	3420	Shady Moist	40°	NW	31° 23.209 N	78° 25.577 E
21	3450	Bouldery	50°	W	31° 23.881 N	78° 21.441 E
22	3480	Shady Moist	50°	NW	31° 20.886 N	78° 26.179 E
23	3500	Shady Moist	40°	NW	31° 23.959 N	78° 21.554 E
24	3501	Moraine	45°	SE	31° 20.967 N	78° 27.303 E
25	3516	Rocky	40°	SE	31° 21.002 N	78° 27.381 E
26	3527	Moraine	45°	SE	31° 21.002 N	78° 27.374 E
27	3650	Shady Moist	40°	N	31° 20.636 N	78° 26.224 E
28	3700	Shady Moist	40°	N	31° 20.515 N	78° 26.267 E
29	3770	Shady Moist	35°	NW	31° 20.389 N	78° 26.334 E
30	3850	Shady Moist	30°	NE	31° 20.167 N	78° 26.411 E
31	4129	Alpine meadow	45°	NE	31° 19.310 N	78° 26.151 E
32	4205	Alpine meadow	25°	N	31° 19.242 N	78° 26.049 E
33	4330	Moraine	30°	NE	31° 18.831 N	78° 25.888 E
34	4500	Alpine meadow	20°	NE	31° 18.347 N	78° 25.648 E

**Table 2:** Taxonomic account of floristic diversity.

Taxonomic group	Families	Genera	Species	Herbs	Shrubs	Trees
Angiosperms	68	190	302	243	39	20
Gymnosperms	4	7	13	—	4	9
Pteridophytes	3	3	5	5	—	—
Total	**75**	**200**	**320**	**248**	**43**	**29**

**Table 3:** Identified communities showing TBA, species richness, densities, and diversity in Sangla Valley.

Communities	SR	TBA (m^2^/ha)	Species richness	Density			Species diversity index (*H*′)		
				Trees (no./ha)	Shrubs (no./ha)	Herbs (no./m^2^)	Trees	Shrubs	Herbs
Trees									
BU	5	11.17	47	480	330	35.16	0.73	2.12	3.17
CD	3	42.41	38	403.33	673.33	33.44	0.57	1.68	3.62
QF	1	14.17	19	340	290	22.08	0	0.4	2.49
PG	3	12.09	47	490	606.29	35.20	0.82	1.16	3.11
PW	6	8.702	96	205	625.83	36.34	0	2.38	4.01
BU-AP	1	14.16	35	440	770	25.03	1.02	1.52	3.16
BU-PW	4	12.28	80	420	327.5	40.36	0.93	2.22	3.69
CD-PS	1	17.98	41	600	1030	42.40	1.28	2.07	2.98
CD-PW	1	21.99	29	400	420	38.20	0.69	0.95	3.07
Shrubs									
HS	2	—	41	—	370	45.39	—	1.63	3.21
RA	2	—	40	—	105	32.56	—	0.96	3.45
RA-SC	2	—	52	—	230	34.80	—	1.15	3.55
SCa-CB-BJ	1	—	34	—	540	48.73	—	1.53	2.87
SCa-LH	1	—	33	—	630	27.70	—	1.43	3.1
Herbs									
PA-AS-BA-AV	1	—	34	—	—	78.95	—	—	2.91

SR = site represented, TBA: total basal area, Ind = individual, BU = *Betula utilis*, CD = *Cedrus deodara*, QF = *Quercus floribunda*, PG = *Pinus gerardiana*, PW = *Pinus wallichiana*, BU-AP = *Betula utilis-Abies pindrow* mixed, BU-PW = *Betula utilis-Pinus wallichiana* mixed, CD-PS = *Cedrus deodara-Picea smithiana*, CD-PW = *Cedrus deodara-Pinus wallichiana* mixed, HS = *Hippophae salicifolia*, RA = *Rhododendron anthopogon*, RA-SC = *Rhododendron anthopogon-Salix caliculata* mixed, SCa-CB-BL = *Spiraea canescens-Cotoneaster bacillaris-Berberis jaeschkeana *mixed, SCa-LH = *Spiraea canescens-Lonicera hypoleuca* mixed, and PA-AS-BA-AV = *Poa alpina-Agrostis stolonifera-Bistorta affinis-Aconitum violaceum*.

**Table 4:** Relative densities of dominant species in various communities.

Communities	Dominant elements		
	Trees	Shrubs	Herbs
**Trees**			
*Betula utilis* D. Don	*B. utilis *D. Don (74.5%), *P. wallichiana *A. B. Jacks.* * (19.9%), and *A. pindrow * (Royle ex D. Don) Royle (5.0%)	*Cassiope fastigiata* (Wall.) D. Don (28.5%), *Rhododendron campanulatum* D. Don (17.6%), and *Rosa webbiana *Wall. ex Royle (9.7%)	*Polygonatum verticillatum* (L.) All. (11.4%), *Geranium wallichianum* D. Don ex Sweet (5.7%),* Fragaria nubicola * (Hook. f.) Lindl. ex Lacaita (5.6%), and* Aconogonum molle * (D. Don) H. Hara (5.4%)
*Cedrus deodara * (Roxb. ex Lamb.) G. Don	*C. deodara * (Roxb. ex Lamb.) G. Don (80.2%),* P. wallichiana *A. B. Jacks. (17.4%), and* A. pindrow * (Royle ex D. Don) Royle (2.5%)	*Rabdosia rugosa * (Wall. ex Benth.) H. Hara (31.3%),* Berberis aristata * (29.8%), and* Cotoneaster bacillaris *Wall. ex Lindl. (7.5%)	*Thalictrum cultratum *Wall. (11.1%)*, F. nubicola * (7.4%), and *Nepeta erecta* (Royle ex Benth.) Benth. (7.4%)
*Quercus floribunda* Lindl. ex A. Camus	*Q. floribunda* Lindl. ex A. Camus (100%)	*R. rugosa * (Wall. ex Benth.) H. Hara (80.1%), *Sorbaria tomentosa * (Lindl.) Rehder (9.1%)	*Cannabis sativa *L. (14.3%)*, Dysphania botrys * (L.) Mosyakin & Clemants (10.9%), and* Tagetes minuta *L. (9.5%)
*Pinus gerardiana* Wall. ex D. Don	*Pinus gerardiana* Wall. ex D. Don (57.14%),* Q. floribunda* Lindl. ex A. Camus (38.8%), and *Olea europaea* L. (4.1%)	*R. rugosa * (Wall. ex Benth.) H. Hara (55.9%),* Desmodium elegans* DC. (18.9%), and* Artemisia maritima *L. (13.2%)	*Conyza sumatrensis* (S. F. Blake) Pruski & G. Sancho (9.4%),* C. sativa *L. (6.8%), and* Chenopodium album *L. (5.4%)
*Pinus wallichiana* A. B. Jacks.	*P. wallichiana *A. B. Jacks. (90.9%),* C. deodara * (Roxb. ex Lamb.) G. Don (8.1%)	*Rosa macrophylla* Lindl. (17.4%),* Berberis jaeschkeana *C. K. Schneid. (14.4%), and* Cotoneaster microphyllus *Wall. ex Lindl. (12.8%)	*Origanum vulgare *L. (6.1%),* Morina longifolia *Wall. (3.6%), and* Medicago lupulina *L. (3.3%)
*B. utilis* D. Don*-A. pindrow *Royle ex D. Don) Royle mixed	*B. utilis* D. Don (43.1%),* A. pindrow * (Royle ex D. Don) Royle (40.9%),and*P. wallichiana *A. B. Jacks. (15.9%)	*C. microphyllus *Wall. ex Lindl. (32.5%),* R. macrophylla * Lindl. (22.0%), and* B. jaeschkeana *C. K. Schneid. (11.7%)	*Cynoglossum wallichii G. Don * (*23.4%*)*, F. nubicola * (Hook. f.) Lindl. ex Lacaita (18.2%), *Trigonella emodi* Benth., and *Lotus corniculatus *L. (4.6% each)
*B. utilis* D. Don*-P. wallichiana* A. B. Jacks. mixed	*B. utilis* D. Don (62.5%),* P. wallichiana *A. B. Jacks. (29.8%), and* A. pindrow * (Royle ex D. Don) Royle (4.3%)	*C. fastigiata* (Wall.) D. Don (33.3%), *Spiraea canescens *D. Don (10.0%), and *Rhododendron anthopogon *D. Don (7.6%)	*Persicaria vivipara* (L.)* Ronse Decr. * (6.8%), *Delphinium cashmerianum* Royle (4.5%), and* Lomatogonium carinthiacum * (Wulfen) Rchb. (3.5%)
*C. deodara * (Roxb. ex Lamb.) G. Don-*Piceasmithiana* (Wall.) Boiss. mixed	*C. deodara * (Roxb. ex Lamb.) G. Don (38.3%), *Picea smithiana* (Wall.) Boiss. (31.6%),* P. wallichiana *A. B. Jacks. (20.0%), and *Juglans regia*L. (10.3)	*Rubusellipticus* Sm. (15.5%),* B. aristata *DC. (14.6%), and* D. elegans *DC. (13.6%)	*F. nubicola * (Hook. f.) Lindl. ex Lacaita (16.1%),* Trifolium pratense *L. (5.2%),* T. cultratum *Wall., and* Chaerophyllum villosum *Wall. & DC. (3.8% each)
*C. deodara * (Roxb. ex Lamb.) G. Don*-P. wallichiana* A. B. Jacks. mixed	*C. deodara * (Roxb. ex Lamb.) G. Don (52.5%),* P. wallichiana *A. B. Jacks. (42.5%), and *P. smithiana* (Wall.) Boiss. (5.0%)	*B. jaeschkeana *C. K. Schneid. (59.52%),* S. canescens *D. Don (21.4%),* R. webbiana *Wall. ex Royle (19.0)	*F. nubicola * (Hook. f.) Lindl. ex Lacaita (8.2%),* T. linearis *Benth. (6.3%), and* T. alpinus *L. (5.5%)
**Shrubs**			
*Hippophae salicifolia *D. Don	—	*Hippophae salicifolia *D. Don (58%),* Sorbaria tomentosa * (Lindl.) Rehder (13.2%), and* Rabdosia rugosa * (Wall. ex Benth.) H. Hara (9.4%)	*Fragaria nubicola * (Hook. f.) Lindl. ex Lacaita (15.6%), *Cannabis sativa *L. (8.3%)*, Polygonum aviculare *L. (6.0%),* *and* Nepeta erecta * (Royle ex Benth.) Benth. (4.5%)
*Rhododendron anthopogon *D. Don	—	*Rhododendron anthopogon *D. Don (57.2%),* Salix caprea *L. (28.6%), and *Cotoneaster microphyllus *Wall. ex Lindl. (14.3%)	*Delphinium cashmerianum *Royle (7.2%),* Rhodiola himalensis * (D. Don) S. H. Fu (5.7%),* Bistorta affinis * (D. Don) Greene (5.3%)*, Lactuca macrorhiza * (Royle) Hook. f. (4.8%), and* Impatiens thomsonii* Hook. f. (4.2%)
*Rhododendron anthopogon *D. Don*-Salix calyculata *Hook. f. ex Andersson mixed	—	*Rhododendron anthopogon *D. Don (45.65%),* Salix calyculata *Hook. f. ex Andersson (36.9%), and* Viburnum cotinifolium *D. Don (8.7%)	*F. nubicola * (Hook. f.) Lindl. ex Lacaita (19.5%),* Bistorta affinis * (D. Don) Greene (10.7%),* Anaphalis triplinervis * (Sims) Sims ex C. B. Clarke (6.7%),* Poa annua *L. (6.2%),* Thalictrum cultratum *Wall. (5.7%), and* Oxyria digyna * (L.) Hill (5.3%)
*Spiraea canescens *D. Don*-Cotoneaster bacillaris *Wall. ex Lindl.-*Berberis jaeschkeana *C. K. Schneid. mixed	—	*Spiraea canescens *D. Don (27.0%),* Cotoneaster bacillaris *Wall. ex Lindl. (11.5%), and* Berberis jaeschkeana *C. K. Schneid. (8.2%)	*Elsholtzia eriostachya * (Benth.) Benth. (17.2%),* Fragaria nubicola * (Hook. f.) Lindl. ex Lacaita (15.4%),* Euphrasia officinalis* L. (7.7%),* Origanum vulgare *L. (7.4%),* *and *Impatiens thomsonii* Hook. f. (3.5%)
*Spiraea canescens *D. Don*-Lonicera hypoleuca *Decne. Mixed	—	*Spiraea canescens *D. Don. (44.4%),* Lonicera hypoleuca *Decne (19.0%), and* Berberis jaeschkeana *C. K. Schneid. (11.1%)	*Bromus japonicus *Thunb. (10.5%),* Cuscuta capitata *Roxb. (9.6%),* Aconogonum molle * (D. Don) H. Hara (7.2%), and* Fragaria nubicola * (Hook. f.) Lindl. ex Lacaita (6.3%)
**Herbs**			
*Poa alpina *L.-*Agrostis stolonifera *L.-*Bistorta affinis * (D. Don) Greene*-Aconitum violaceum *Jacquem. ex Stapf mixed	—	—	*Poa alpina *L. (25.33%),* Agrostis stolonifera *L. (13.2%),* Bistorta affinis * (D. Don) Greene (4.6%),* Aconitum violaceum *Jacquem. ex Stapf (4.6%), and *Heracleum thomsonii *C. B. Clarke (4.3%)
